# In numbers we trust?

**DOI:** 10.7554/eLife.02791

**Published:** 2014-04-01

**Authors:** Eve Marder

**Affiliations:** Department of Biology and the Volen National Center for Complex Systems, Brandeis University, Waltham, United Statesmarder@brandeis.edu

**Keywords:** living science, research assessment, scientific publishing, publishing, article-level metric, eLife

## Abstract

Scientists go to great lengths to ensure that data are collected and analysed properly, so why, asks **Eve Marder**, do they apply different standards to data about the number of times research papers have been cited and viewed?

We are all scientists and we believe in measuring, counting, and calculating. We measure the length of cilia, we monitor the reaction times of humans, and we would count the exact number of whales in the ocean, if we could. We learn how to estimate when we can’t measure and count, and a big part of becoming a scientist is learning how to evaluate the accuracy and reliability of our data. We worry about calibrating our instruments, we obsess about the resolution of our microscopes, and we stress about false positives and false negatives. We calculate statistics, either correctly or incorrectly, in hope of gaining confidence that we are capturing some truth through our measurements. As scientists, there are remarkably few instances when we would think a single measurement or count is ‘the truth’.

**Figure 1:**
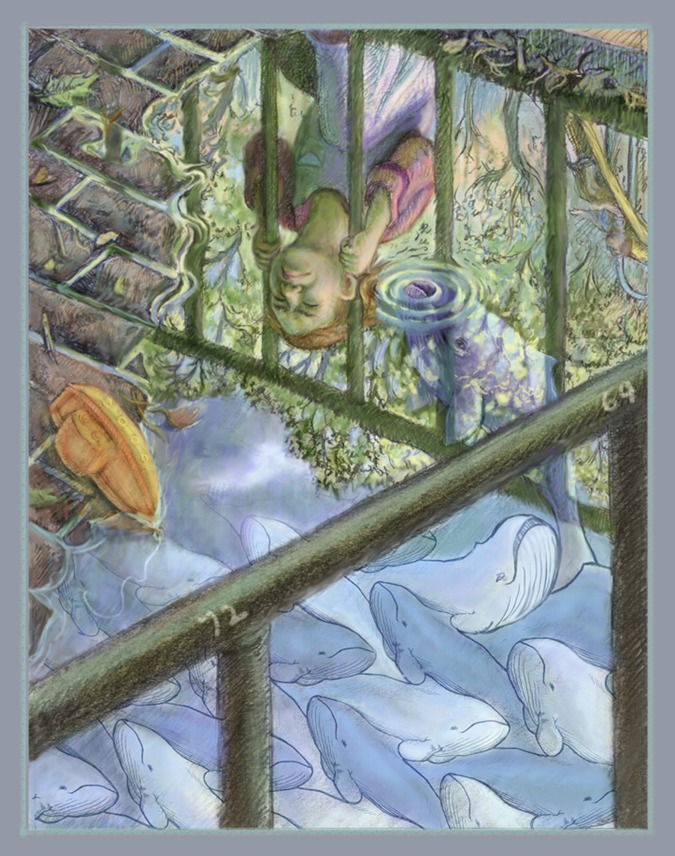
Scientists should focus on the science in research papers, not the number of times the paper has been cited or viewed.

ILLUSTRATION: BEN MARDER.

Many, many editorials and opinion pieces have been published about the right and wrong metrics to use to evaluate journals, university departments or individual scientists. Journal impact factors, the numbers of citations, h-indexes, page views, downloads and various other measures have their advocates and their critics but—with a few exceptions—the conversation about these metrics has involved an innocent and blind assumption that they have been calculated in a reliable fashion. Otherwise, no sane person could imagine using any or all of these metrics to inform decisions about hiring or funding!

I recently saw a CV from an investigator who used Google Scholar as the source of his h-index and the number of citations for each of his papers. I had never looked at Google Scholar, so on a whim I checked my own h-index and the number of citations for some of my papers on both Google Scholar and Web of Science. (I was avoiding doing a particularly annoying academic task, so this was an amusing waste of time). To my surprise, my h-index was 72 on Google Scholar and 64 on Web of Science, a not inconsequential difference. Then I compared the number of times that some of my papers had been cited. I found that one paper (Hooper and Marder, 1987) had 167 citations in Google Scholar and 161 in Web of Science, which was a difference of just a few per cent. But then I discovered that another paper (Marder and Calabrese, 1996) had 894 citations on Google Scholar and only 636 citations on Web of Science, which was a difference of about 30%!

*eLife*, like many journals, reports the number of times each article in the journal has been viewed (along with the number of times it has been cited according to Scopus, HighWire and Google Scholar) as another way of assessing the impact of individual articles. In the past 18 months I have published five ‘Living Science’ essays in *eLife*. Every few months prurient interest and vanity have caused me to look at number of views each of these pieces has attracted. Imagine my confusion when, recently, I discovered that the number of views for two of them had dropped substantially during the past several months! I still don’t know if the old values or new values are correct, or why the number of views has decreased, but it does give me pause in believing the reliability of such numbers, not only in *eLife*, but as posted by all journals.

But then I started thinking. Why should we have ever trusted those numbers? What kinds of systematic, or field specific, or journal specific, or author specific errors are likely in citation data? How is it possible that thousands of careful scientists who spend months and years checking and rechecking their own data, would blithely and blindly accept these numbers? Is a journal impact factor of 3.82 different from one of 4.3, even though there are some review panels who treat them as if they are different and make decisions about promotions accordingly? How many of us would not ask questions of investigators in their own fields about how the data were collected, analysed and verified? And yet how many of us have the foggiest idea how exactly the numbers of citations for publications have been collected, and what verification procedures are used?

How many of us have the foggiest idea how exactly the numbers of citations for publications have been collected?

One might argue that the actual numbers do not matter, and it is only orders of magnitude at issue. If so, why even bother with the numbers? At the limit, the numbers just validate what we already know. Someone like Linda Buck or Eric Kandel has more citations than any assistant professor. But we don’t need indexes to tell us that Nobel Prize winners have had impact and influence on their fields, and that scientists who have only been in the field for 10 years have had less cumulative effect.

When I was three years old I got my head stuck between the bars of a playground in Central Park because I was curious if my head was larger or smaller than the distance between the bars. As I stood there unable to get my head out of the bars, I felt very stupid, as I realised I could have measured the distance between the bars by holding my hands apart, without getting stuck. It was my first real experience of feeling truly self-consciously dumb. Many years later I feel equally stupid for believing that the numbers that have become so important to so many careers are reliable. I have never believed that these numbers are wisely used in assessments, but now I know there is all the more reason to forget all of these indexes and metrics and to focus on the science.
